# Engineered extracellular vesicles carrying *let-7a-5p* for alleviating inflammation in acute lung injury

**DOI:** 10.1186/s12929-024-01019-4

**Published:** 2024-03-19

**Authors:** Sin-Yu Chen, Yi-Ling Chen, Po-Chen Li, Tai-Shan Cheng, Yeh-Shiu Chu, Yi-Shan Shen, Hsin-Tung Chen, Wei-Ni Tsai, Chien-Ling Huang, Martin Sieber, Yuan-Chieh Yeh, Hsiao-Sheng Liu, Chi-Ling Chiang, Chih-Hung Chang, Andrew S. Lee, Yen-Han Tseng, Ly James Lee, Hsiu-Jung Liao, Hon-Kan Yip, Chi-Ying F. Huang

**Affiliations:** 1 Institute of Biopharmaceutical Sciences, College of Pharmaceutical Sciences, National Yang Ming Chiao Tung University, Taipei, 112304 Taiwan; 2 Division of Cardiology, Department of Internal Medicine, Kaohsiung Chang Gung Memorial Hospital and Chang Gung University College of Medicine, Kaohsiung, 833401 Taiwan; 3 Institute for Translational Research in Biomedicine, Kaohsiung Chang Gung Memorial Hospital, Kaohsiung, 833401 Taiwan; 4 Department of Orthopedic Surgery, Far Eastern Memorial Hospital, New Taipei City, 220216 Taiwan; 5 Brain Research Center, National Yang Ming Chiao Tung University, Taipei, 112304 Taiwan; 6 Department of Biomedical Engineering, National Taiwan University, Taipei, 106319 Taiwan; 7 Bionet Therapeutics, Taipei, 114065 Taiwan; 8 Department of Traditional Chinese Medicine, Chang Gung Memorial Hospital, Keelung, 204201 Taiwan; 9 Program in Molecular Medicine, College of Life Sciences, National Yang Ming Chiao Tung University, Taipei, 112304 Taiwan; 10 Department of Microbiology and Immunology, College of Medicine, National Cheng Kung University, Tainan, 701401 Taiwan; 11 Center for Cancer Research, College of Medicine, Kaohsiung Medical University, Kaohsiung, 807378 Taiwan; 12 Teaching and Research Center, Kaohsiung Municipal Siaogang Hospital, Kaohsiung Medical University Hospital, Kaohsiung Medical University, Kaohsiung, 812015 Taiwan; 13 Department of Chemical and Biomolecular Engineering, The Ohio State University, Columbus, OH 43210 USA; 14 Comprehensive Cancer Center, College of Medicine, The Ohio State University, Columbus, OH 43210 USA; 15 Graduate School of Biotechnology and Bioengineering, Yuan Ze University, Taoyuan, 320315 Taiwan; 16 Spot Biosystems Ltd., Palo Alto, CA 94305 USA; 17 Department of Chest Medicine, Taipei Veterans General Hospital, Taipei, 112201 Taiwan; 18 Department of Medical Research, Far Eastern Memorial Hospital, New Taipei City, 220216 Taiwan; 19 Center for Shockwave Medicine and Tissue Engineering, Kaohsiung Chang Gung Memorial Hospital, Kaohsiung, 833401 Taiwan; 20 Department of Nursing, Asia University, Taichung, 413305 Taiwan; 21 Department of Medical Research, China Medical University Hospital, China Medical University, Taichung, 404328 Taiwan; 22 Department of Biochemistry, School of Medicine, Kaohsiung Medical University, Kaohsiung, 807378 Taiwan

**Keywords:** *Let-7a-5p*, Extracellular vesicles, Mesenchymal stem cells, Cellular nanoporation, Anti-inflammation, Acute lung injury

## Abstract

**Background:**

Acute lung injury (ALI) is a life-threatening respiratory condition characterized by severe inflammation and lung tissue damage, frequently causing rapid respiratory failure and long-term complications. The microRNA *let-7a-5p* is involved in the progression of lung injury, inflammation, and fibrosis by regulating immune cell activation and cytokine production. This study aims to use an innovative cellular electroporation platform to generate extracellular vesicles (EVs) carring *let-7a-5p* (EV-*let-7a-5p*) derived from transfected Wharton’s jelly-mesenchymal stem cells (WJ-MSCs) as a potential gene therapy for ALI.

**Methods:**

A cellular nanoporation (CNP) method was used to induce the production and release of EV-*let-7a-5p* from WJ-MSCs transfected with the relevant plasmid DNA. EV-*let-7a-5p* in the conditioned medium were isolated using a tangential flow filtration (TFF) system. EV characterization followed the minimal consensus guidelines outlined by the International Society for Extracellular Vesicles. We conducted a thorough set of therapeutic assessments, including the antifibrotic effects using a transforming growth factor beta (TGF-β)-induced cell model, the modulation effects on macrophage polarization, and the influence of EV-*let-7a-5p* in a rat model of hyperoxia-induced ALI.

**Results:**

The CNP platform significantly increased EV secretion from transfected WJ-MSCs, and the encapsulated *let-7a-5p* in engineered EVs was markedly higher than that in untreated WJ-MSCs. These EV-*let-7a-5p* did not influence cell proliferation and effectively mitigated the TGF-β-induced fibrotic phenotype by downregulating SMAD2/3 phosphorylation in LL29 cells. Furthermore, EV-*let-7a-5p* regulated M2-like macrophage activation in an inflammatory microenvironment and significantly induced interleukin (IL)-10 secretion, demonstrating their modulatory effect on inflammation. Administering EVs from untreated WJ-MSCs slightly improved lung function and increased *let-7a-5p* expression in plasma in the hyperoxia-induced ALI rat model. In comparison, EV-*let-7a-5p* significantly reduced macrophage infiltration and collagen deposition while increasing IL-10 expression, causing a substantial improvement in lung function.

**Conclusion:**

This study reveals that the use of the CNP platform to stimulate and transfect WJ-MSCs could generate an abundance of *let-7a-5p*-enriched EVs, which underscores the therapeutic potential in countering inflammatory responses, fibrotic activation, and hyperoxia-induced lung injury. These results provide potential avenues for developing innovative therapeutic approaches for more effective interventions in ALI.

**Supplementary Information:**

The online version contains supplementary material available at 10.1186/s12929-024-01019-4.

## Background

Acute lung injury (ALI) is a severe and life-threatening lung condition characterized by acute inflammation and damage to lung tissue, causing impaired oxygen exchange [1]. Lung injury progresses to fibrosis by activating profibrotic pathways and persistent inflammation, accumulating lung scar tissue over time [2, 3]. Currently, all types of lung injury have no universally approved drug, and even medications, such as nintedanib and pirfenidone, which are approved for idiopathic pulmonary fibrosis, need to comprehensively address lung conditions [4, 5]. The coronavirus disease-2019 (COVID-19) pandemic has caused more acute respiratory distress syndrome (ARDS) cases and long COVID-related lung symptoms [6]. Consequently, developing therapeutics that halt inflammation and fibrosis is crucial in the progression of lung injury.

Stem cell therapy has emerged as an innovative approach to address ALI and its associated fibrotic changes. Previous studies have revealed the potential of human umbilical cord mesenchymal stem cells (HUC-MSCs) to improve tissue repair and reduce inflammation through their regenerative and immunomodulatory properties [7–9]. The therapeutic effects of MSCs result from their paracrine actions, specifically those of extracellular vesicles (EVs). Hence, stem cell-derived secretomes or conditioned mediums, which contain many EVs, have gained attention for their therapeutic benefits [10, 11]. EVs represent a recent advancement in regenerative medicine, providing a promising avenue for treating ALI [12]. Various cell types release these small, membrane-bound vesicles that serve as intercellular communication carriers. They facilitate the transfer of vital bioactive molecules, including miRNAs, proteins, and growth factors, which play pivotal roles in modulating cellular responses, regulating inflammation, and promoting tissue repair. The use of these nanosized EVs as drug delivery systems has demonstrated the capability to prolong circulation time and improve cellular uptake compared with lipid nanoparticles (LNPs) [13]. Harnessing the regenerative potential of EVs constitutes a groundbreaking approach that can transform ALI management and its associated fibrotic complications.

RNA therapeutics have demonstrated significant potential for addressing respiratory diseases [14–17]. Recent advancements have highlighted the efficacy of small interfering RNAs (siRNAs) as nucleic acid-based treatments for respiratory disorders, including ALI/ARDS [18, 19]. The distinct advantages of RNA-based therapy lie in its ability to design interventions through bioinformatics and achieve heightened potency [20]. Both siRNAs and microRNAs (miRNAs) function by targeting the critical pathways associated with these conditions. A pivotal breakthrough in the clinical translation of RNA therapy was achieved by developing effective delivery strategies, which accelerated the attainment of favorable and rapid outcomes in ALI/ARDS treatment.

Our prior investigations have highlighted the potential of honeysuckle (*Lonicera japonica*) in mitigating cytokine storms induced by severe acute respiratory syndrome coronavirus 2 (SARS-CoV-2) [21, 22]. Honeysuckle holds particular significance as a primary component in more than half of the patented anti-inflammatory (anti-heat toxin) traditional Chinese medicine formulations. Furthermore, it ranks among the most frequently prescribed herbs for treating COVID-19 [23, 24]. Several active chemical constituents of honeysuckle have been identified for their direct binding to protein targets, thereby demonstrating immunomodulatory effects [25]. Moreover, our research has revealed that honeysuckle stimulates the host *let-7a-5p*, which possesses potent antiviral and anti-inflammatory properties [21, 26] that have appeared as a critical player in ALI. *Let-7a-5p* has been associated with inhibiting viral replication, thereby providing a unique avenue for addressing viral infection-induced ALI. Previous research has revealed that host *let-7a-5p*, induced by honeysuckle, effectively hinders viral replication by targeting specific regions within the RNA genomes of viruses [22, 26]. Furthermore, *let-7a-5p* downregulation has been observed in patients with ALI [27, 28], indicating its potential role in disease pathogenesis.

Therefore, we introduce a method for engineering EVs using a new cellular nanoporation (CNP) platform [29]. We aimed to combine two promising therapeutic strategies, including MSC-derived EVs and therapeutic miRNA, to produce therapeutic EVs enriched with *let-7a-5p*. This approach elucidates the profitable RNA-based therapeutic strategy using engineered EVs to treat ALI. Our research reveals that *let-7a-5p*-enriched EVs (EV-*let-7a-5p*) counteracted the fibrotic phenotype induced by TGF-β and promoted M2 polarization in an in vitro system. Furthermore, EV-*let-7a-5p* attenuated hyperoxic ALI in rats. These compelling results collectively emphasize the potential of *let-7a-5p*-enriched EVs as a therapeutic tool to mitigate inflammation, facilitate tissue recovery, and hinder fibrotic processes in ALI.

## Materials and methods

### Cell culture

Bionet Therapeutics, Taiwan, kindly provided WJ-MSCs for EV production and all subsequent in vitro and in vivo experiments. Cell analysis through flow cytometry was conducted to confirm the presence of specific markers (for MSCs: CD105^+^, CD44^+^, CD29^+^, CD90^+^, CD73^+^, CD13^+^, CD45^−^, CD14^−^, CD34^−^, CD31^−^, CD19^−^, and HLA-DR^−^) before their use. WJ-MSCs were cultured in Minimum Essential Medium α (MEM α, GIBCO, Gaithersburg, MD, USA) supplemented with 5% UltraGRO Advanced (AventaCell, Atlanta, USA), 1% penicillin, and streptomycin (GIBCO, Gaithersburg, MD, USA) at 37 °C with 5% CO_2_. LL29 cells were purchased from the American Type Culture Collection and maintained in Ham’s F12K medium (GIBCO, Gaithersburg, MD, USA) supplemented with 10% fetal bovine serum (FBS), 1% penicillin, and streptomycin at 37 °C in 5% CO_2_.

### TGF-β stimulation to induce the fibrotic phenotype in LL29 cells

TGF-β stimulation is crucial in inducing a fibrotic phenotype, primarily by activating fibroblasts to transform into myofibroblasts, which are key in producing excess extracellular matrix (ECM) components such as collagen. This process also inhibits ECM degradation and potentially induces epithelial–mesenchymal transition (EMT), further contributing to tissue fibrosis. This cell model was used to mimic lung injury to fibrosis progression compared with animal studies. LL29 cells were cultured in a medium containing 10% FBS and treated with 10 ng/mL of TGF-β to facilitate the fibroblast-to-myofibroblast transition and induce a fibrotic phenotype as an in vitro model.

### Cell transfection

A single layer of WJ-MSCs (~ 300,000 cells) was spread on a 24 mm^2^ CNP track-etched membrane surface for overnight incubation for CNP (Spot Biosystems). Cells were transfected with the *let-7a-5p* plasmid suspended in phosphate-buffered saline (PBS) buffer through pore channels on a track-etched membrane using a 125 V electric field administered in 10 pulses, each lasting 10 ms, with intervals of 0.1 s between pulses under an electric field by the Xcell System (Bio-rad). The plasmid pcDNA3-pri-*let-7a* was a gift from Narry Kim (Addgene plasmid #51377; http://n2t.net/addgene:51377; RRID: Addgene_51377)[30].

### Collection and purification of EVs from donor cells

The cells were cultured in a serum-enriched culture medium. The medium was then replaced with serum-free culture medium after washing the cells three times with PBS, and the cells were subsequently incubated for 24 h following CNP. Next, the conditioned medium (CM) underwent centrifugation at 300 × *g* for 5 min to remove cells and at 2000 × *g* for 20 min to eliminate cell debris. A 0.22-μm PES membrane filter was used to sterilize the CM. EVs were collected and diafiltrated using a tangential flow filtration (TFF) system with a peristaltic pump (Lefo Science Co., Ltd) with hollow fiber (MidiKros). The size distribution and concentration of EV samples were identified using nanoparticle tracking analysis (NTA) conducted with a NanoSight NS300 instrument (Malvern Instruments, MA, US).

### Negative staining of EVs by transmission electron microscopy (TEM)

The isolated WJ-MSC EVs were visualized using an electron microscope (JEM-1400plus, JEOL Ltd.) with negative staining. EVs were fixed with 100 μL of 4% paraformaldehyde (PFA) for 5 min. The EV suspension was loaded 5 μL on the formvar/carbon film-coated 200 mesh copper EM grids and incubated for 1 min. The filtered 1% uranyl acetate (UA) solution was placed on the surface of the EM grid for 5 min. The excess UA solution was then removed from the grid by contacting the grid edge with filter paper. A drop of water was quickly rinsed from the grid to remove the excess staining solution. The grids were placed in an EM grid box and placed in a dry box for future observation by TEM at 100 kV.

### Analysis of the EV surface composition with ExoView

We used the ExoView tetraspanin kit (NanoView Biosciences, Boston, MA) with immobilized CD9, CD63, and CD81 antibodies on silicon dioxide chips to capture EVs. An ExoView™ R200 imaging system was used to scan EV samples.

### Protein quantification and exosome identification by Western blotting

Cell and EV samples were lysed using a lysis buffer (Thermo Fisher Scientific) and homogenized using an ultrasonicator (UP-50H, Hielscher GmbH). Protein quantification was performed using the BCA assay kit (Thermo Fisher Scientific). Lysates were diluted with 4X Laemmli Sample Buffer and loaded with equal amounts of total protein into SDS-PAGE gels. The total protein was transferred onto a PVDF membrane with a pore size of 0.45 μm and blocked with 5% Non-fat milk for 1 h. The membrane was then probed with the following antibodies: anti-calnexin (1:1000; 2679, Cell Signaling), anti-CD9 (1:1000; ab236630, Abcam), anti-CD63 (1:1000; ab216130, Abcam), anti-CD81 (1:1000; sc-166029, Santa Cruz), anti-αSMA (1:1000; 19245, Cell Signaling), anti-fibronectin (1:1000; GTX112794, GeneTex), anti-Smad2/3 (1:1000; 8685, Cell Signaling), anti-phospho-Smad2/3 (1:1000; 8828, Cell Signaling), anti-COL1A1 (1:1000; ab34710, Abcam), anti-GAPDH (1:5000; GTX100118, GeneTex), anti-IL-10 (1:1500; Sigma-Aldrich, St. Louis, MO, USA), anti-TGF-β (1:1000, #ab50036 Abcam, anti-rabbit), and anti-β-actin (1:1000; #MAB1501, Sigma-Aldrich). The Immobilon ECL Ultra Western HRP Substrate (Millipore) and a GE LAS-4000 were used to detect protein bands.

### Relative and absolute quantitative analysis of *let-7a-5p* expression using quantitative reverse transcription polymerase chain reaction (RT-qPCR)

A commercially available kit to purify cell-free total RNA, primarily miRNA and other small RNA (Qiagen’s miRNeasy Serum/Plasma Kit), was used for RNA isolation. Subsequently, the miRCURY LNA RT Kit was used for the RT of RNA. Real-time PCR was conducted with cDNA using the miRCURY LNA miRNA SYBR^®^ Green master mix and commercially available primers (*Let-7a-5p* primer: QIAGEN, QIA-339306). Cel-miR-39 (cel-39) (Qiagen), which serves as a spike-in control, was introduced into each EV sample at known quantities to evaluate the efficiency of the RT reaction. Synthetic *let-7a-5p* (5’-UGAGGUAGUAGGUUGUAUAGUU-3’) was also purchased as a standard for calculating the miRNA copies.

### Cell viability assay

Cell viability was assessed via the SRB assay. Cells were seeded at a density of 6000 cells/well in a 96-well plate. After 48 h of treatment with the indicated treatments, the cells were fixed with 10% trichloroacetic acid (TCA, Sigma, cat. SI-T6399-250G). The fixed cells were washed twice with PBS and stained with sulforhodamine B sodium salt dye (SRB dye, Sigma, cat. S1402) for 30 min. The stained cells were washed three times to remove unbound dye using 1% (v/v) acetic acid. The protein-bound dye was dissolved in a 10 mM Tris-base (Amresco, cat. CPT-0826) solution for absorbance (510 nm) measurement using a multimode microplate reader (Infinite 200 PRO). The relative cell proliferation rate was then compared with the control group.

### EV uptake assay

EVs were labeled using CellVue dye (Sigma-Aldrich). Hence, 2.0 × 10^10^ EVs were incubated with 2 mM of CellVue dye for 10 min. After labeling, the EVs were washed twice using a 10 kDa filter (Millipore, Amicon 10 K) to remove excess dye. Subsequently, CellVue dye-labeled EVs were administered at a concentration of 1 × 10^9^ particles for the uptake experiment. An end-point test was performed by allowing the uptake of EVs with living cells for 30 min and acquiring dual-color confocal images for the entire cell volume (Z-axis = 20 μm). The z-stack images were projected onto one Z plane, individual cell contours were subdivided by cell mask signals, dots inside the cells were binarized, and their numbers were counted using Metamorph and Image J. Fluorescence intensity was measured using confocal microscopy and Total Internal Reflection Fluorescence microscopy.

### Human M2 macrophage induction by flow cytometry and enzyme-linked immunosorbent assay (ELISA)

Human M2 macrophage induction and subsequent FACS analysis involved isolating peripheral mononuclear cells from healthy donor blood using standard density gradient centrifugation with Ficoll-Paque. CD14^+^ cells were then isolated from these peripheral mononuclear cells using high-gradient magnetic sorting. CD14^+^ monocytes were cultured for seven days in a complete Roswell Park Memorial Institute-1640 medium. They were supplemented with 10 ng/mL of human macrophage colony-stimulating factor for the first six days, followed by an additional day of culturing with 20 ng/mL of IL-4 to induce an M2-like macrophage phenotype.

Monoclonal mouse antihuman antibodies were used to stain specific markers on human macrophages for FACS analysis. Monoclonal mouse antihuman CD163 (BioLegend, Catalog no. 333610) and CD206 (mannose receptor, MR) antibodies (Invitrogen, Catalog no. 48-2069-42) were used to label M2 macrophages. Cells were suspended in PBS at 1 × 10^5^ cells/mL, and 5% BSA buffer was used to block nonspecific antigens. Analysis was performed using a CytoFLEX flow cytometer (Beckman Coulter), with nonspecific mouse immunoglobulin as an isotype control.

ELISA was used to quantify IL-10, IL-1RN, TNF-α, IL-6, and IL-1β production levels. The concentrations of IL-10 (BioLegend, Catalog no. 430604), IL-1RN (R&D Systems, Catalog no. DRA00B), and CCL22 (R&D Systems, Catalog no. DY336) were measured in the supernatants of M2-like macrophages. Commercially available ELISA kits (R&D Systems, Human IL-1RN, Catalog no. DY280; Human IL-10, Catalog no. DY217B) were used following the manufacturer’s instructions.

### Characterization of M1 macrophages by flow cytometry and ELISA

The isolation process for M0 was identical to the differentiation of M2 macrophages from human peripheral blood mononuclear cells (PBMCs). M1 polarization was achieved by treating the cells with interferon-γ (IFN-γ, 10 ng/mL, BioLegend, Catalog no. 570204) and lipopolysaccharides from *Escherichia coli* (LPS, 50 ng/mL, Sigma-Aldrich, Catalog no. L2880) for 48 h. Antihuman iNOS (BioLegend, Catalog no. 696805) and HLA-DR (BioLegend, Catalog no. 307657) antibodies were used to mark M1 macrophages for flow cytometry analysis. Moreover, ELISA was used to measure the concentrations of TNF-α (BioLegend, Catalog no. 430204), IL-6 (R&D Systems, Catalog no. DY206), and IL-1β (BioLegend, Catalog no. 437004) in the supernatants of M1-like macrophages.

### Animal grouping for ALI induction and treatment strategy

The animals were housed in an Association for Assessment and Accreditation of Laboratory Animal Care International-approved animal facility with a controlled temperature (24 ± 1 ℃) and 12:12 light/dark cycle. This study used pathogen-free, adult male Sprague–Dawley rats weighing 325–350 g (Charles River Technology, BioLASCO Taiwan Co. Ltd., Taiwan). An ALI experimental model was developed when pure oxygen (100% oxygen) was continuously administered to rats for 72 h (all hyperoxia induction groups). We conducted a pilot study to validate the ALI model before the actual study [31]. Rats were randomly divided into six groups: normal controls (Sham), ALI (Vehicle), ALI + EV-Ctrl (2 × 10^10^ particles), ALI + EV-Ctrl (1 × 10^11^ particles), ALI + EV-CNP (2 × 10^10^ particles), and ALI + EV-*let-7a-5p* (2 × 10^10^ particles). EVs were intravenously injected into the rats at 48 and 72 h under 100% oxygen exposure.

### Arterial oxygen saturation determination

Arterial blood was sampled from the carotid artery for blood gas analysis after 48 and 72 h of 100% oxygen exposure to investigate the therapeutic effects of EV-CNP and EV-*let-7a-5p* treatment on arterial oxygen saturation (SaO_2_). After arterial blood sampling, rats were euthanized and their lungs were harvested.

### Histological assessment of lung injury

The lung specimens were sectioned at 5 μm for light microscopy. Hematoxylin and eosin (H&E) staining was performed to estimate the number of alveolar sacs in a blinded fashion, as previously reported [31]. Three lung sections from each rat were analyzed, and three randomly selected high-power fields (HPFs; 100 ×) were investigated in each section. The mean number per HPF for each animal was then determined by summing all numbers divided by 9. The extent of the crowded area, which was a region of thickened septa in lung parenchyma associated with partial or complete alveolar collapse on H&E-stained sections, was also performed in a blinded fashion. The following scoring system [31] was adopted: 0 = no detectable crowded area; 1 ≤ 15% of the crowded area; 2 = 15–25% of the crowded area; 3 = 25–50% of the crowded area; 4 = 50–75% of the crowded area; and 5 ≥ 75–100% of crowded area/HPF.

### Immunocytochemistry (IHC) and immunofluorescence (IF) staining

The dehydrated para sections were first treated with 3% H_2_O_2_ for 30 min and incubated with Immuno-Block reagent (BioSB) for 30 min at 25 ℃. Sections were then incubated with primary antibodies specifically against F4/80 (1:100; sc-377009, Santa Cruz Biotechnology, CA, USA) and CD68 (1:500; ab31630, Abcam, UK), whereas sections incubated with irrelevant antibodies served as controls. Three sections of lung specimens were analyzed in each rat. Three randomly selected HPFs (400 × for IHC and IF studies) were analyzed in each section for quantification. The mean number of positively stained cells per HPF for each animal was then determined by dividing all the numbers by 9.

### Statistics and data analysis

Data are reported as means ± standard error of the mean for at least three independent experiments unless stated otherwise. GraphPad Prism 10 (GraphPad Software, San Diego, CA) was used for graphs and statistical analyses. A two-tailed unpaired Student’s *t*-test for comparison of two treatment groups or a one-way ANOVA analysis of variance to compare multiple treatment groups were used for statistical analysis of the results. A *p*-value of < 0.05 was considered significant (*p < 0.05, **p < 0.01, ***p < 0.001, ns = non-significant).

## Results

### Production and characterization of engineered EVs

**Fig. 1:**
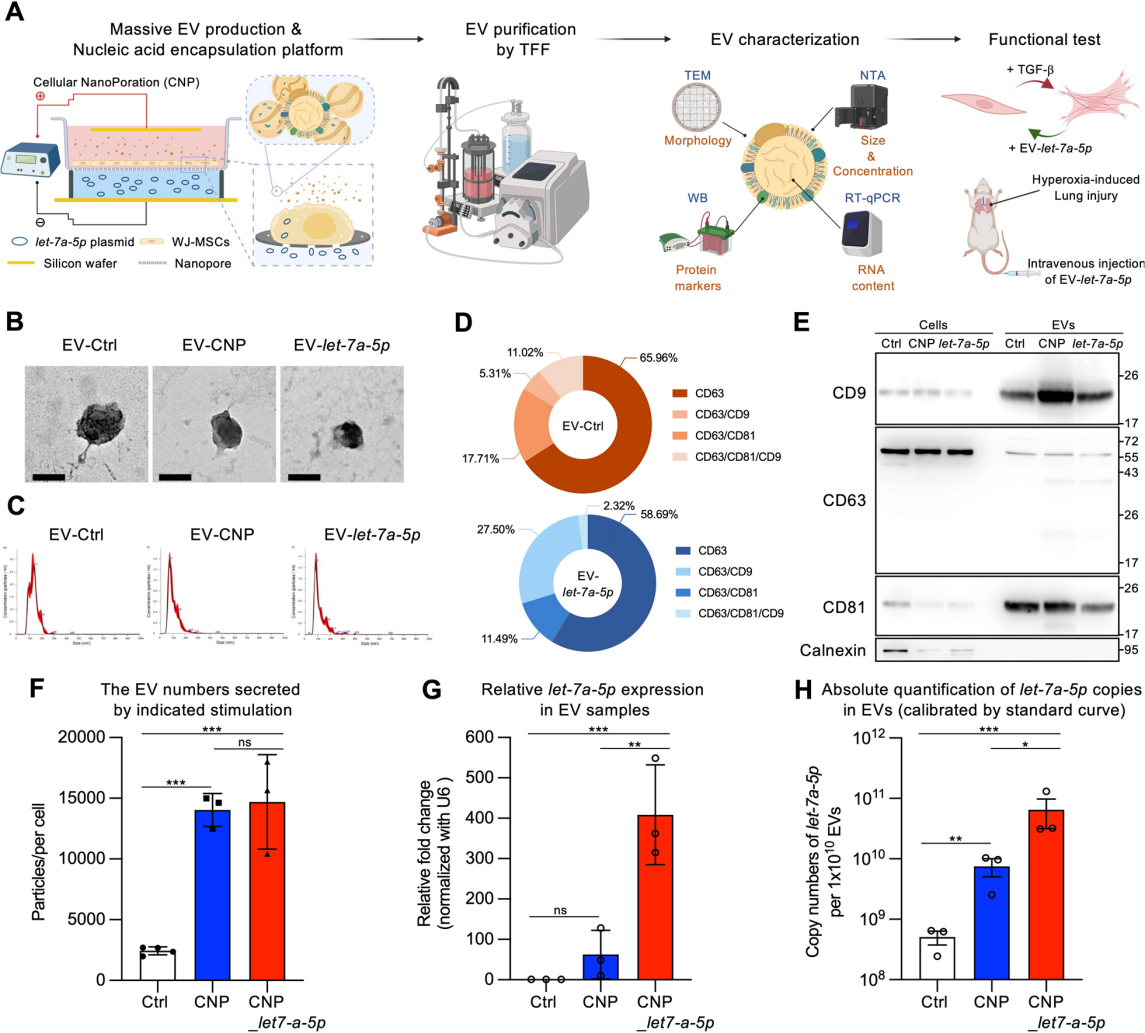
Production and characterization of engineered EVs. **A** Schematic flowchart illustrating the engineered EV production and characterization. Donor cells on the track-etched membrane are transfected with plasmid DNA from below the insert through high-electric field strength in pores. CNP improves the release of EVs carrying therapeutic RNA cargoes transcribed from plasmids. The supernatant containing EVs was then purified by the TFF method. TEM, WB, NTA, and RT-qPCR were used to investigate the EV characterization before all functional tests and in vivo experiments. The illustration was created with BioRender.com. **B** TEM images displayed the morphology of EVs. Scale bar = 100 nm. **C** Measurement of three groups of EVs using NTA. **D** Representative pie charts obtained from the ExoView chip illustrated the co-localized percentage of total particles detected with various CD markers, including CD63, CD63/CD9, CD63/CD81, or CD63/CD81/CD9. These charts correspond to different capture spots/EVs. **E** Western blot analysis of exosomal CD markers in total cell lysate or EVs, with Calnexin as the negative control for EVs. **F** Quantification of particle numbers secreted by donor cells with the indicated stimulation. The MSCs processed by the CNP platform significantly improved the EV production rate per cell compared to non-electroporated cells. **G** Detection of the relative expression of *let-7a-5p* using RT-qPCR. **H** Absolute quantification of *let-7a-5p* copies using standard curve calibration. For **B–H,** the images are representative of n = 3 biologically independent experiments. Data were presented as mean ± standard error of the mean. For **F–H**, the data were analyzed by two-tailed unpaired Student’s *t*-test. (*p < 0.05, **p < 0.01, ***p < 0.001, ns = non-significant)

Our experimental process started by developing a CNP platform [32] to produce *let-7a-5p*-enriched EVs (EV-*let-7a-5p*) for potential therapeutic applications in ALI. We focused on using clinical-grade MSCs as the source of EVs because they have demonstrated therapeutic potential in human lung injury treatment [7, 8]. These WJ-MSCs were characterized by positive expression of surface markers and demonstrated the ability to differentiate into osteocytes, adipocytes, and chondrocytes in vitro (Additional file 1: Figure S1). WJ-MSCs were cultured on a track-etched membrane and then transfected with a *let-7a-5p* plasmid using electroporation to produce EV-*let-7a-5p*. After a 24-h incubation period, serum-free CM was collected for EV isolation (Fig. 1A). Three distinct conditions were used to obtain CM: EV-Ctrl (naive EV derived from WJ-MSCs), EV-CNP (CNP with PBS as vehicle control), and EV-*let-7a-5p* (CNP with *let-7a-5p* plasmid) for comparison. The collected CM underwent two low-speed spins and a 0.22-µm filtration step, followed by TFF system purification (Fig. 1A). TEM and NTA revealed a diverse population of EVs with diameters ranging from approximately 50 to 200 nm (Fig. 1B and C). These vesicles demonstrated characteristic morphological attributes typically associated with small EVs (sEVs). Next, we used ExoView to confirm the colocalization of specific markers (Fig. 1D and Additional file 1: Figure S2), and Western blotting was used to validate the presence of exosomal markers (CD9, CD63, and CD81) on these EVs, which are characteristic of exosomes (Fig. 1E). Importantly, our analysis revealed the absence of the nonexosomal marker Calnexin (Fig. 1E). We observed a significant increase in EV production with the CNP platform. The average concentrations of isolated EVs in three independent batches were as follows: EV-Ctrl at 1.38 × 10^8^ ± 2.25 × 10^7^ particles/mL, EV-CNP at 8.07 × 10^8^ ± 5.78 × 10^7^ particles/mL, and EV-*let-7a-5p* at 8.74 × 10^8^ ± 1.65 × 10^8^ particles/mL (Additional file 1: Table S1). Notably, the CNP platform resulted in an approximately sixfold increase in the rate of EV production per cell compared with non-electroporated cells (Fig. 1F). Surprisingly, the EVs from the EV-CNP group, which underwent electroporation without *the let-7a-5p* plasmid, also demonstrated an increased level of *let-7a-5p* content within the EVs (Fig. 1G). Moreover, the CNP platform demonstrated remarkable efficiency in producing EVs encapsulating *let-7a-5p*, with an approximately 400-fold increase in *let-7a-5p* content within EVs (Fig. 1G). We precisely quantified the copy numbers of *let-7a-5p* contained within 1 × 10^10^ EVs, which is a crucial step for quality control in therapeutic applications. To achieve this, we used synthetic *let-7a-5p* to develop a standard curve, enabling us to accurately identify the quantity of *let-7a-5p* within the EVs (Additional file 1: Figure S3). The *let-7a-5p* copy numbers within 1 × 10^10^ EVs in each group were as follows: EV-Ctrl had 5.05 × 10^8^ copies, EV-CNP had 7.51 × 10^9^ copies, and EV-*let-7a-5p* had 6.46 × 10^10^ copies (Fig. 1H). Collectively, these results demonstrate that the CNP platform significantly improves EV secretion and *let-7a-5p* encapsulation into EVs, particularly exosomes, indicating that our platform holds promise for using EVs as effective carriers for miRNA encapsulation.

### EV-*let-7a-5p* suppresses TGF-β-induced fibrotic phenotype in vitro

**Fig. 2:**
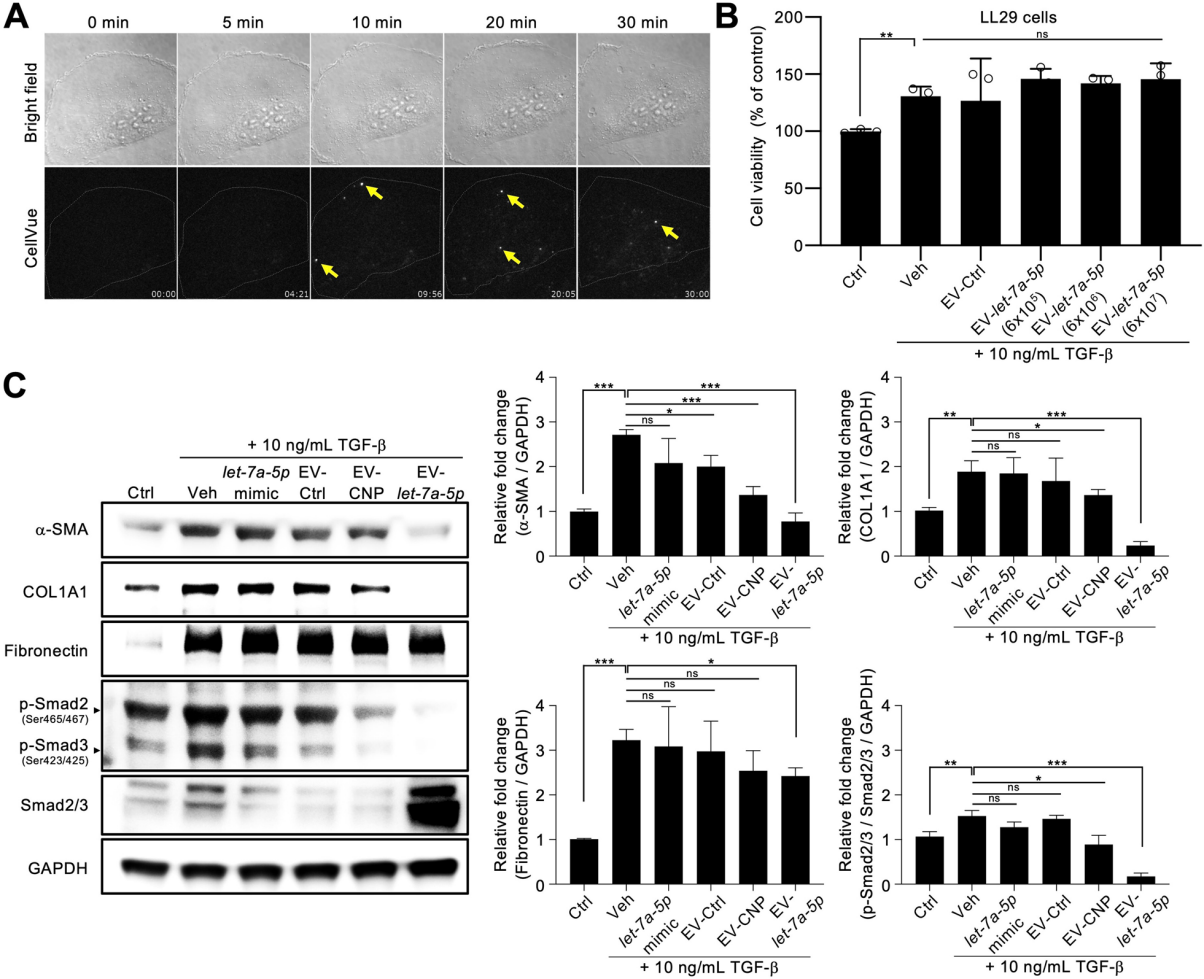
EV-*let-7a-5p* suppresses TGF-β-induced fibrotic phenotype in vitro. **A** Fluorescent images of stained cells were taken by Total Internal Reflection Fluorescence microscopy (TIRFM, Nikon) and quantitated for co-localization by Imaris software (BITPLANE, Oxford Instruments, Zurich, Switzerland). EVs were labeled with CellVue Claret and incubated with BEAS-2B cells at 37 °C for 30 min. **B** Cell viability was assessed using the SRB assay after 48 h of the indicated treatment. The cell density is 6000 cells per well treated with three concentrations of 6 × 10^5^, 6 × 10^6^, and 6 × 10^7^ EVs. **C** The fibrotic phenotype of LL29 cells was induced by TGF-β treatment. It demonstrated advanced fibrosis-related markers, characterized by increased Smad2/3 (phospho-Smad2/3) activation and elevated α-SMA, COL1A1, and fibronectin expression. Lipid nanoparticles, loaded with 10 pg of synthetic *let-7a-5p* and packaged using lipofectamine 3000, serve as a EV-*let-7a-5p* mimic (*let-7a-5p* mimic). This fibrotic response was effectively mitigated by EV-*let-7a-5p* (10,000 EVs per cell). Data were presented as mean ± standard deviation. The images are representative of n = 3 biologically independent experiments. For **C**, the data were analyzed by two-tailed unpaired Student’s *t*-test. (*p < 0.05, **p < 0.01, ***p < 0.001, ns = non-significant)

We used a coadministration approach, introducing TGF-β simultaneously with EVs during LL29 cell cultivation, to evaluate the impact of EV-*let-7a-5p* on the TGF-β-induced transformation of LL29 fibroblasts into myofibroblasts. Initially, we investigated the internalization of EVs by TGF-β-induced LL29 cells. An internalization assay using fluorescently labeled EVs monitored by an epifluorescence microscope revealed that cells can uptake engineered EVs (Additional file 1: Figure S4). We then further tracked the single EV by total internal reflection fluorescent microscopy. However, we adopted an alternative approach using BEAS-2B lung epithelial cells and A549 cells to demonstrate the internalization of EVs in lung-associated cell lines because of the substantial laser-induced autofluorescence exhibited by LL29 cells (Fig. 2A, Additional file 1: Figure S4). Notably, EV-*let-7a-5p* demonstrated internalization capability in BEAS-2B cells. Furthermore, EV-*let-7a-5p* demonstrated no significant effects on cell viability during the 48-h incubation period (Fig. 2B).

TGF-β is extensively used in the pathogenesis of fibrosis in lung injury [33]. With TGF-β treatment, LL29 cells were first induced into a fibrotic phenotype with increased α-SMA, COL1A1, and fibronectin expressions. Further administration of EV-*let-7a-5p* (10,000 EVs per cell) for 48 h strongly suppressed the TGF-β-induced fibrotic phenotype by reducing the α-SMA and COL1A1 expression (Fig. 2C). Additionally, phosphorylation of Smad3 is essential in the TGF-β signaling pathway [34, 35]. Upon TGF-β exposure, phospho-Smad3 expression increased in LL29 cells, whereas EV-*let-7a-5p* treatment inhibited TGF-β-dependent phosphorylation of Smad3 (Fig. 2C). This observation indicates that EV-*let-7a-5p* suppresses TGF-β signaling by attenuating Smad3 activation. A previously published method was used for bioinformatics-based analysis of *let-7a-5p* [21], and the results confirm our experimental findings. A total of 990 predicted targets were retrieved from miRDB, including COL1A1, Fibronectin (FNDC3A and FNDC3B), SMAD2, IL10, and TAB2 (TGF-β activated kinase 1 binding protein 2) (Additional file 1: Table S2). Pathway analysis using CPDB revealed that *let-7a-5p* may regulate fibrosis, collagen synthesis pathways, and inflammatory-related pathways (PI3K-Akt and MAPK signaling pathway) (Additional file 1: Table S3). These data indicate that *let-7a-5p*-enriched EVs inhibit TGF-β-induced fibrotic phenotype.

### EV-*let-7a-5p* drive M0 macrophages toward M2-like macrophage polarization

**Fig. 3:**
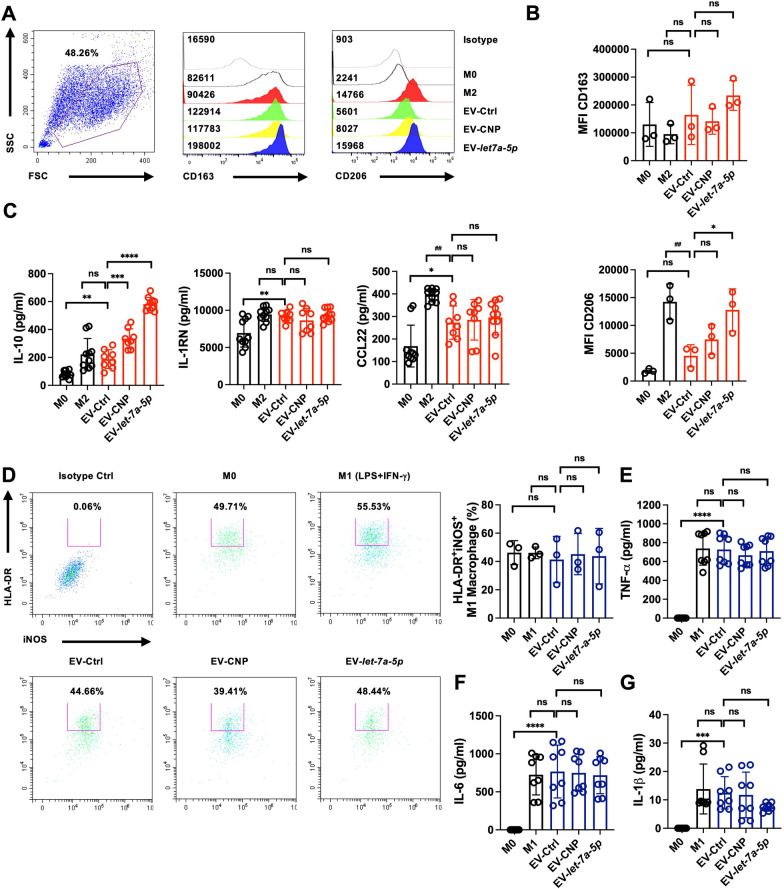
The EV-*let-7a-5p* predominantly induces differentiation of human M2-like macrophages, not M1-like macrophages. **A** Flow cytometry data illustrating the M2-like macrophage populations expressing CD206 marker following a 7-day treatment with EV-*let-7a-5p* (1 × 10^9^ particles/mL). **B** Quantifying the mean fluorescence intensity (MFI) of CD163 and CD206 by EV-*let-7a-5p*. **C** EV-*let-7a-5p* improved the release of IL-10 in M2-like macrophages, as assessed through ELISA. **D** A representative flow cytometry dot plot illustrated the expression of iNOS and HLA-DR in human M1 macrophages after treatment with EV-*let-7a-5p*, with LPS and IFN-γ treatment as positive controls. Quantification of the induction of iNOS^+^HLA-DR^+^ cell populations by EV-*let-7a-5p*. **E–G** The effect of EV-*let-7a-5p* on TNF-α, IL-6, and IL-1β production in M1-like macrophages evaluated through ELISA, revealing no significant changes. The results are representative of n = 3 biologically independent experiments. Statistical analysis used one-way ANOVA analysis of variance, indicating significance (*p < 0.05, **p < 0.01, ***p < 0.001, ns = non-significant) for comparisons between treatment groups and either M2/M1 skewing or other specified groups

We investigated the role of EV-*let-7a-5p* in promoting M2-like macrophage polarization. Our results revealed that EV-*let-7a-5p* induction led to significant changes in human M2-like macrophages, as assessed by flow cytometry for CD206 and CD163 (Fig. 3A and B) and through the measurement of the anti-inflammatory cytokines IL-10 and IL-1RN and the chemokine CCL22 by ELISA (Fig. 3C). IL-4 stimulation was used as a positive control to induce M2 macrophages (Fig. 3D). Thus, the markers associated with M2-like macrophages in polarized human CD14^+^ monocytes demonstrated a notable increase in IL-10 production due to EV-*let-7a-5p* induction but not IL-1RN and CCL22. We then explored whether EV-*let-7a-5p* directly affected the suppression of M1-like macrophage phenotypes. The expression of HLA-DR and iNOS was upregulated when M0 macrophages were exposed to both LPS and IFN-γ, indicating a tendency toward an inflammatory macrophage phenotype. Similarly, EV-*let-7a-5p* did not decrease M1-like macrophage polarization (Fig. 3D) or TNF-α, IL-6, and IL-1β secretion (Fig. 3E-G). This implies that EV-*let-7a-5p* had no direct influence and did not affect the production of inflammatory cytokines when introduced from M0 macrophages to M1-like macrophages.

### WJMSC-EVs reduce lung injury and improve survival in ALI

**Fig. 4:**
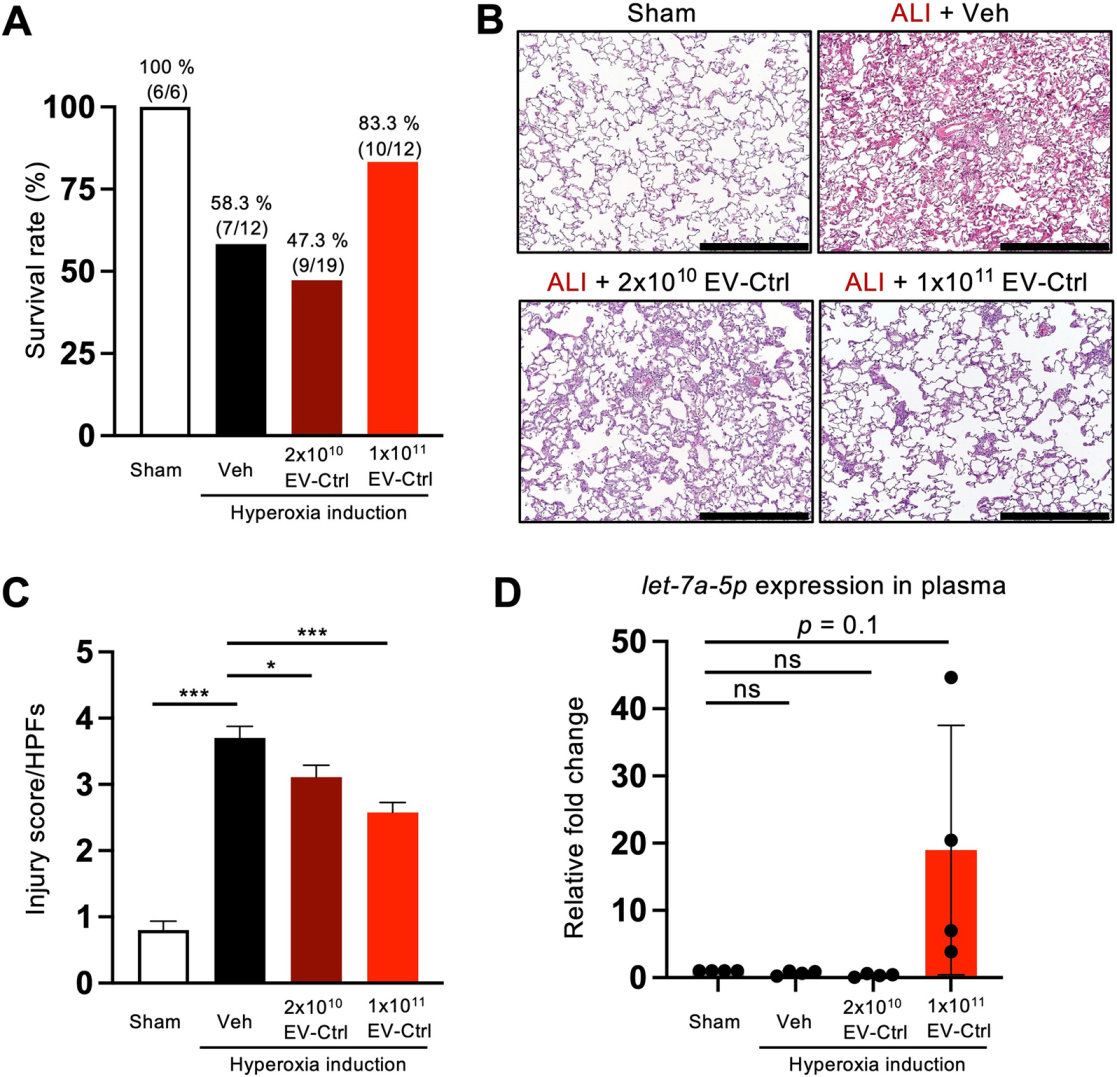
WJMSC-EVs reduce lung injury and improve the survival of ALI. **A** Survival rate of sham, ALI rats, ALI treated with 2 × 10^10^ EV-Ctrl, or ALI treated with 1 × 10^11^ EV-Ctrl. **B** Evaluation of lung histology by hematoxylin and eosin staining. Scale bars = 500 μm. (n of sham, ALI, 2 × 10^10^ EV-Ctrl, 1 × 10^11^ EV-Ctrl = 6, 7, 9 and 10 rats, respectively). **C** Lung injury scores were assessed. **D** The measurement of *let-7a-5p* expression in the plasma using qRT-PCR. Data were presented as mean ± standard deviation. Statistical analyses were performed using a two-tailed unpaired Student’s *t*-test. (*p < 0.05, **p < 0.01, ***p < 0.001, ns = non-significant)

We developed a hyperoxia-induced lung injury rat model to mimic ALI to address the therapeutic potential of WJ-MSC-derived EVs. Intravenous injection of EVs was performed on day 2 after hyperoxia induction to investigate lung injury improvement. The survival rate was 100% in a control rat (6/6), 58.3% (7/12) in the vehicle group (hyperoxia induction only), 47.3% (9/19) in the 2 × 10^10^ EV-Ctrl treated rat, and 83.3% (10/12) in 1 × 10^11^ EV-Ctrl treated rat. Compared with control rats, the survival rate was higher in EV-Ctrl with 1 × 10^11^ particles than in the vehicle group with a significant survival rate in EV-treated rats (Fig. 4A). Examination of H&E-stained lung sections revealed that the scoring of lung parenchymal crowding was lowest in the vehicle group and was significantly rescued by EV treatment (Fig. 4B and C). We then examined the expression level of *let-7a-5p* in the plasma, and we revealed that *let-7a-5p* was increased in the plasma compared with the other groups (Fig. 4D). In line with in vitro experiments, WJ-MSC-derived EVs exerted more potent anti-inflammatory effects on hyperoxia-induced lung injury than the vehicle group.

### EV-*let-7a-5p* reduces inflammation and improves ALI rats

**Fig. 5:**
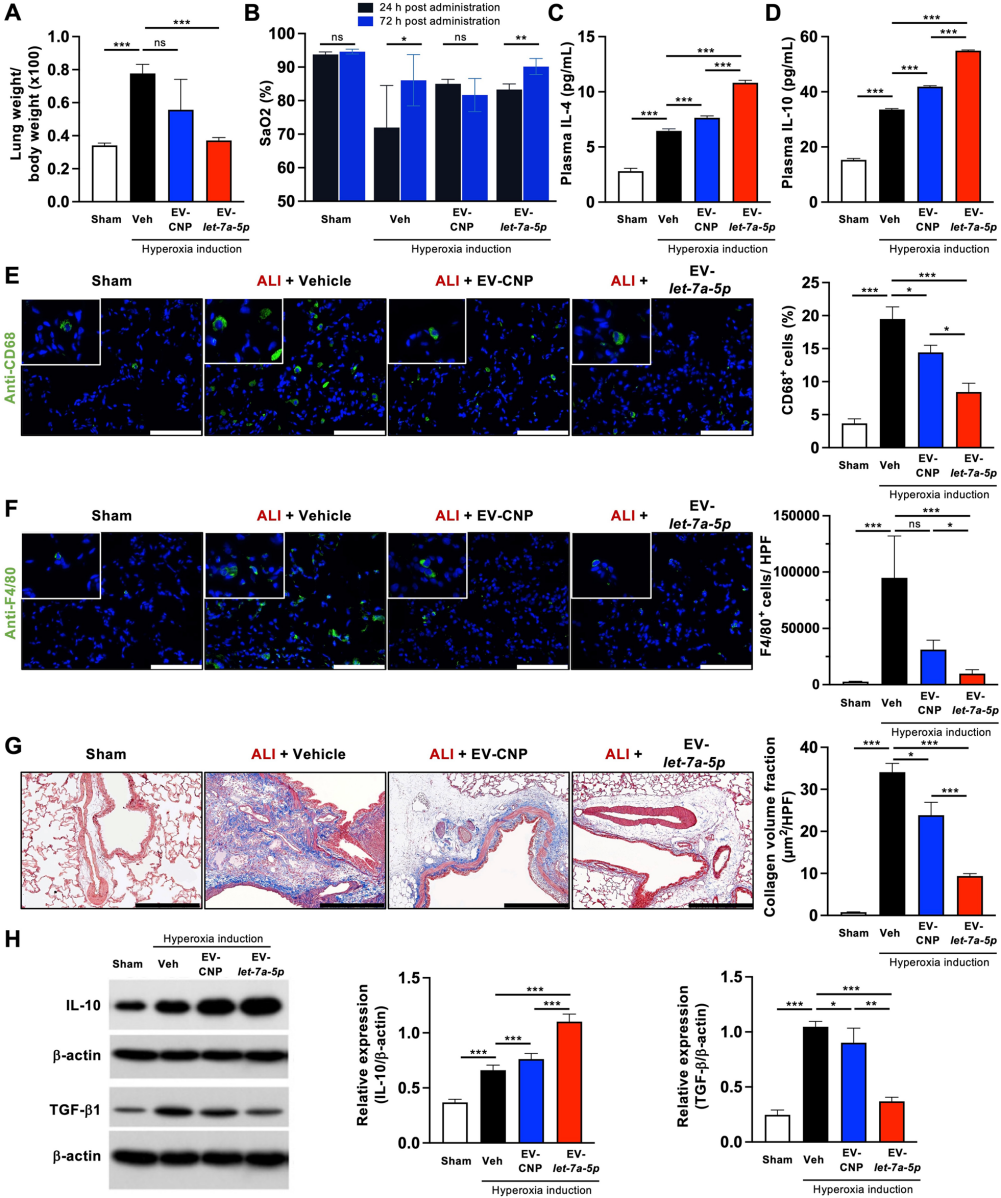
EV-*let-7a-5p* ameliorates inflammation and improve ALI rat condition. **A** Lung weight-to-body weight ratio assessment. **B** Arterial oxygen saturation (SaO_2_) of the carotid artery was measured by Waters Medical Systems Oxicom 3000A. The plasma levels of IL-4 (**C**) and IL-10 (**D**) were detected by ELISA. **E, F** CD68^+^ and F4/80^+^ cells in the lung parenchyma were detected by immunofluorescence. Representative images show monocytes/macrophages (F4/80^+^, green; CD68^+^, green). Nuclei were counterstained with DAPI (blue). Scale bar = 100 μm. **G** Collagen deposition (blue) in the lung parenchyma was detected by Masson trichrome staining. Scale bar = 100 μm. **H** The protein expressions of IL-10 and TGF-β were detected by Western blot. β-actin as the internal. Statistical analyses were performed using a Student’s *t*-test. Sham: healthy rat (n = 6 rats). Veh: ALI treated with PBS (n = 7 rats). EV-CNP: ALI treated with EV-CNP (n = 3 rats). EV-*let-7a-5p*: ALI treated with *let-7a-5p* enriched EVs (n = 6 rats). Data were presented as mean ± standard deviation. The data were analyzed by two-tailed unpaired Student’s t-test. (*p < 0.05, **p < 0.01, ***p < 0.001, ns = non-significant)

We assessed the potential of EV-*let-7a-5p* in ameliorating hyperoxia-induced lung injury in a rat model. Specifically, we focused on the role of EV-CNP and EV-*let-7a-5p*, as our results indicated that EV-Ctrl did not cause significant improvements (Fig. 4). Hence, we administered 2 × 10^10^ EVs via intravenous injection two days after hyperoxia induction and evaluated various lung injury parameters. First, we examined the ratio of total lung weight-to-body weight. Notably, the vehicle group demonstrated the highest weight ratio, indicating severe inflammation. However, this ratio could be restored through EV-CNP and EV-*let-7a-5p* treatment, with the most significant improvement observed in the EV-*let-7a-5p* group (Fig. 5A). Additionally, we evaluated oxygen saturation (SaO_2_) in the carotid arterial blood using pairwise comparisons (Student’s *t*-test). The results revealed the following comparisons: (1) Sham, 24 h vs. 72 h, *p* = 0.076; (2) Vehicle, 24 h vs. 72 h, *p* = 0.019; (3) EV-CNP, 24 h vs. 72 h, *p* = 0.123; and (4) EV-*let-7a-5p*, 24 h vs. 72 h, *p* < 0.0001 (Fig. 5B). Notably, EV-*let-7a-5p* treatment significantly improved SaO_2_ levels compared with the other groups. Furthermore, we conducted quantitative ELISA analysis of IL-4 and IL-10 in plasma, which are two well-established anti-inflammatory factors. The results indicated increased anti-inflammatory factors in EV-CNP and EV-*let-7a-5p-*treated rats (Fig. 5C and D). We performed IF staining for F4/80^+^ and CD68^+^ cells in the lung to further explore the reduction of inflammation (Fig. 5E and F). These markers are associated with monocytes/macrophages and inflammation. Rats treated with EV-CNP and EV-*let-7a-5p* demonstrated significantly lower levels of monocyte/macrophage infiltration and collagen deposition (Fig. 5E–G). Additionally, we observed increased protein expression of IL-10 and reduced expression of TGF-β in rat lung tissue treated with EV-CNP and EV-*let-7a-5p* (Fig. 5H). Together, we successfully revealed the therapeutic potential of *let-7a-5p*-enriched EVs to decrease inflammation and profibrotic effects in the lung and plasma.

## Discussion

COVID-19 has highlighted the urgent need for effective treatments for ALI/ARDS, which exhibited high morbidity and mortality rates and lacked optimal therapies. Early trials have revealed promise in the use of MSCs and their derivatives to treat ALI/ARDS [36]. These therapies possess antimicrobial, anti-inflammatory, and regenerative properties. Using MSC secretory derivatives, such as EVs, could replicate these benefits and address safety concerns associated with MSC administration. EVs can be developed for inhalation or injection. The meticulously designed process described in this study, involving a CNP platform, ultimately generated *let-7a-5p-*enriched EVs, as confirmed by qPCR analysis, indicating the specific cargo of the *let-7a-5p* miRNA. EV-*let-7a-5p* hold significant promise as potential therapeutics for ALI. Combining clinical-grade WJ-MSCs and non-endocytic delivery of plasmid DNA by the CNP platform for highly effective cell transfection and EV secretion highlights a novel avenue for developing therapeutic interventions for ALI treatment.

Various studies reported approximately 100–400 nucleotides as the average length of RNA in exosomes [37–41], indicating that most exosomal RNAs are generally too short to carry protein-coding information. The miRNA content in MSC exosomes is enriched up to 10 times more than in the cells themselves because miRNAs constitute approximately 0.01% of total cellular RNA by weight [42, 43]. These results emphasize the significance of miRNAs in mediating the functions of MSC-derived EVs. However, notably, a single MSC-derived EV contains approximately 1.3 copies of pre-miRNA molecules, which raises questions about the number of EVs required to deliver sufficient miRNA molecules to elicit a cellular response and induce physiological effects [43, 44]. Therefore, the CNP platform offers an efficient means of producing specific miRNA-enriched EVs. This process involves transecting specific miRNA-coding plasmids, allowing targeted enrichment of specific miRNAs within the resulting EVs. In this study, we selected *let-7a-5p* as a representative therapeutic RNA to reveal its potential to generate engineered EVs for treating ALI. *Let-7a-5p* is a well-supported choice for transfection to enrich EVs because of its multifaceted therapeutic potential (Additional file 1: Table S2 and S3). It has shown promise in inhibiting viral replication, indicating its potential for addressing viral-induced ALI. Furthermore, its reduced expression in patients with ALIs indicates a role in disease pathogenesis [22, 26–28]. *Let-7a-5p* has demonstrated antifibrotic properties by inhibiting hepatic stellate cell activation through the TGF-β/SMAD signaling pathway [45] and reducing fibrotic pathways by suppressing ERα expression [46]. Moreover, the immunomodulatory effects of *let-7a-5p*, which regulate inflammatory responses via the RAS-MAPK pathway [47], further improve its appeal in various disease contexts, making it a promising candidate for targeted EV-based therapies. Cationic LNPs are highly effective for lung delivery via intravenous (IV) injection, but EVs are expected to outperform them. This is because the cationic lipids in LNPs promote inflammation. Moreover, EVs transport complementary miRNAs, which may further improve their performance.

Our results reveal that the CNP platform efficiently encapsulated *let-7a-5p* within EVs and increased the EV production rate (Fig. 1F–H). Interestingly, ExoView assessment revealed differences in exosome subpopulation percentages, distinguished by axosomal CD markers, after processing MSCs using the CNP platform. The percentage of CD9-positive EVs increased from 16.33% (EV-Ctrl) to 29.82% (EV-*let-7a-5p*) (Fig. 1D). This observation was also confirmed by Western blot analysis, which revealed that EV-*let-7a-5p* had higher CD9 expression levels than EV-Ctrl (Fig. 1E). These results indicate that CNP alters cellular EV and exosome production mechanisms to promote EV production. A change is that cell electroporation by CNP causes rapid cell membrane damage and recovery, causing the CD63 endosome to redirect to the plasma membrane. This process increases the CD63/CD9 coexisting subpopulation amounts and may encapsulate mature miRNA within EVs.

Our results reveal that MSC cells processed through the CNP platform demonstrated an increase in *let-7a-5p* loading in EVs even without *let-7a-5p* plasmid transfection (Fig. 1F). These results indicate that the CNP platform causes nonspecific incorporation of endogenous miRNA into EVs, contingent on the cellular miRNA levels. In this study, the enrichment of either endogenous or exogenous *let-7a-5p* within EVs led to enhanced therapeutic effects on ALI. Notably, previous reports have highlighted the role of Ago2 in the sorting of *let-7a-5p* into exosomes [48, 49]. Furthermore, KRAS–MEK–ERK pathway activation contributes to Ago2 accumulation within exosomes [49, 50]. Additionally, K^+^ channel modulation influences Ago2 expression. In contrast, Ago2 binds to the promoter regions of genes encoding calcium-activated potassium channel 3, potassium channel subfamily K member 1, and voltage-gated potassium channel 2, thereby significantly upregulating the expression of these genes [51]. Our CNP platform uses the nanochannel electroporation technique, which has elevated intracellular calcium levels, modifies the open probability of ion channels, and transiently (< 1 s) increases the temperature at the cell face, inducing HSP90 and HSP70 expression [29, 32, 52]. Given these effects, the CNP platform may affect the KRAS–MEK–ERK pathway and ion channels to regulate Ago2 in electroporate MSCs, thereby influencing *let-7a-5p* encapsulation into EVs.

Recently, interest in natural plant-derived products that exhibit potential therapeutic and preventive effects on inflammation and tissue fibrosis has been increasing [53]. This heightened attention is due to their immunomodulatory effects and specific pharmacological activities [54]. We focused on using WJ-MSCs as a carrier to investigate the immunomodulatory function of *let-7a-5p*. We used a novel approach by loading *let-7a-5p* miRNA to investigate its potential in modulating inflammation-induced resident lung fibrosis. Our results revealed that EV-*let-7a-5p* can induce human M2-like macrophage polarization while not affecting M1-like cells (Fig. 3). This leads us to hypothesize that EV-*let-7a-5p* may regulate crosstalk with immune cells, thereby alleviating aberrant inflammation in the tissue-resident microenvironment and providing relief to tissues damaged by long-term inflammation. In summary, these results reveal that *let-7a-5p*-enriched EVs modulate the induction of M2-like macrophage differentiation.

The COVID-19 pandemic caused widespread infections [55]. We used human PBMCs in our in vitro system to investigate the effects of EV-*let-7a-5p* in regulating the polarization of M1-like and M2-like cells. Interestingly, we revealed that human M1-like and M2-like polarization could be influenced by the presence of the infected SARS-CoV-2. Notably, the basal levels of M1-like and M2-like polarization varied significantly among uninfected individuals, those undergoing infection with SARS-CoV-2, and individuals infected with the virus from distinct donors (data not shown). This observation indicates that SARS-CoV-2 may alter immune responses in both innate and adaptive cells by EV-*let-7a-5p*. Further exploration is required to dissect the detailed mechanisms involved in this interaction.

Notably, the p38 MAPK-STAT3 axis is a fundamental pathway governing the initiation, propagation, and resolution of the inflammatory macrophage response, including LPS-induced M1 polarization [56]. The inhibition of alveolar macrophage p38 MAPK expression effectively hindered STAT3 phosphorylation, thereby decreasing the production of the inflammatory cytokine TNF-α, a key player in the pathogenesis of inflammatory lung disease [57]. Furthermore, blockade of this signaling pathway demonstrated an inhibitory effect on proinflammatory M1-type macrophage polarization. It reduced the secretion of proinflammatory factors, including iNOS, IL-1, IL-6, and TNF-α [58]. The overexpression of *let-7a-5p* suppresses the MAPK/ERK signaling pathway [59, 60]. Additionally, MSC exosomes have promoted M2-like macrophage polarization, possibly through the transfer of axosomal miRNAs and activation of AKT-dependent signaling pathways via adenosine receptors A2A and A2B [61–64]. Thus, EV-*let-7a-5p* provide a dual advantage by simultaneously suppressing the MAPK-mediated regulation of STAT3 phosphorylation and activating the AKT pathway, which can be improved to influence macrophage polarization and modulate the inflammatory immune response. Besides its effectiveness in treating lung injury and fibrosis, *let-7a-5p* has demonstrated potential antifibrotic effects in liver fibrosis [45, 65]. *Let-7a-5p* overexpression decreased mRNA levels of α-SMA, COL1A1, and COL1A4, markers of hepatic stellate cell (HSC) activation, indicating its suppressive effect on HSCs. Additionally, *let-7a-5p* transfection reduced HSC viability and triggered apoptosis, with Western blot analysis indicating its inhibitory action through the TGF-β/SMAD signaling pathway [45]. Therefore, in addition to our current study, it provides new insights into the mechanisms underlying the antifibrotic effects of *let-7a-5p*.

However, this study has some limitations. In vivo*,* the limitations of our animal model of ALI include the challenge of conducting lung function tests. Similarly, in clinical settings, physicians face difficulties performing lung function tests on critically ill patients in the Intensive Care Unit (ICU) suffering from acute lung injury.Thus, we evaluated the therapeutic function from EVs derived from WJ-MSCs (EV-Ctrl) through the survival rate of rats and lung injury score. Interestingly, we observed an increased *let-7a-5p* expression after treating the rats with 1 × 10^11^ EV-Ctrl. A previous study revealed a downregulated *let-7a-5p* expression in patients with ARDS, which indicated that *let-7a-5p* plays a pivotal role in inflammatory regulation [28]. Additionally, a previous study validated a reduced *let-7a-5p* in acute-on-chronic liver failure (ACLF) mice, and MSC treatment significantly upregulates *let-7a-5p* expression in primary hepatocytes of ACLF mice [66]. Thus, we used CNPs to overexpress *let-7a-5p* in EVs to exhibit the feasibility of using EV-*let-7a-5p* as a therapeutic approach. Our results indicated that higher levels of encapsulated *let-7a-5p* within EVs improve therapeutic efficacy, potentially allowing for lower EV doses while maintaining therapeutic benefits (Fig. 5). However, the dual nature of *let-7a-5p*’s impact should be considered. Downregulated *let-7a-5p* expression in lung tissue, circulation, and exosomes has been related to ARDS [28, 67, 68], thereby potentially contributing to an exaggerated immune response. However, excessively high *let-7a-5p* expression has been associated with toxicity, with a 20-fold overexpression of *let-7a-5p* resulting in liver damage and dysfunction [69]. Additionally, elevated *let-7a-5p* levels in airway smooth muscle cells can reduce lung compliance by adversely affecting stiffness and force generation, thereby presenting a contradictory aspect in lung injury treatment [70]. *Let-7* is the first miRNA identified in the nematode *Caenorhabditis elegans* and humans, and diverse *let-7a* targets have been reported, including *ras* proto-oncogene and viral RNA genomes. We previously revealed that a traditional Chinese herb, “honeysuckle,” could increase the level of endogenous *let-7a-5p*, suppressing the replication of dengue virus, enterovirus 71, and SARS-CoV-2 [21, 22, 26]. Another study has revealed the successful use of polyethylene glycol (PEG) nanoparticles to deliver *let-7*, which effectively suppresses tumor burden without causing animal toxicity [71]. Altogether, the above results indicate that EV-*let-7a-5p* may also significantly affect other diseases. However, these results collectively emphasize the need for precise regulation of *let-7a-5p* expression to achieve therapeutic efficacy with high safety. Further research is required to identify the optimal dosage that ensures safety and therapeutic benefits.

## Conclusion

**Fig. 6:**
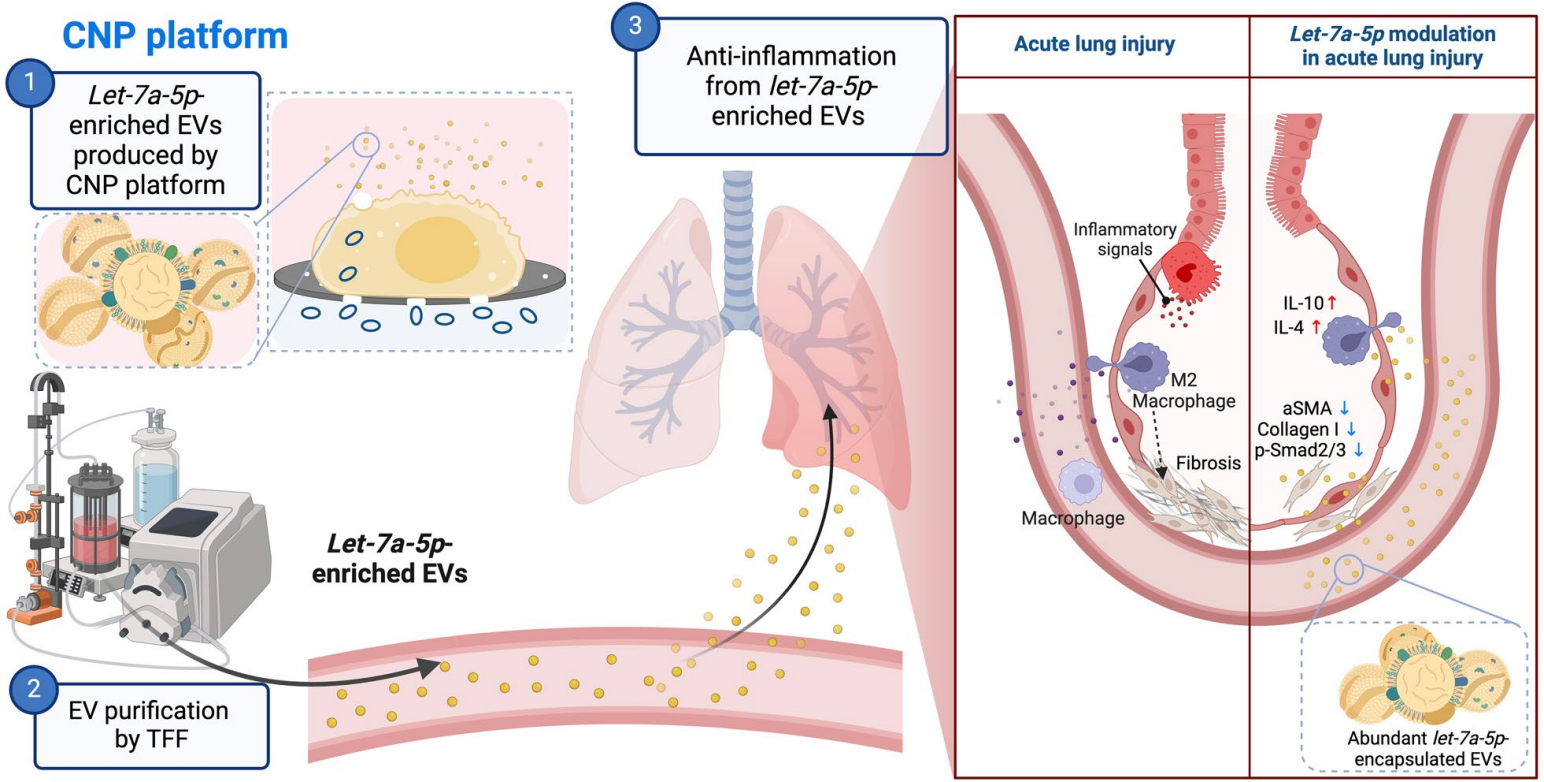
Efficient encapsulation of *let-7a-5p* in MSCs-derived EVs via the CNP platform for acute lung injury treatment. Our results from the CNP platform, cell model, and rat model strongly confirm the following mechanism: The CNP platform provides an efficient method for producing engineered miRNA-enriched EVs with high specificity. These EV-*let-7a-5p* effectively attenuate Smad2/3 activation induced by TGF-β. Additionally, our observations indicate that EV-*let-7a-5p* regulate the induction of M2 macrophages in response to a fibrotic microenvironment resulting from pulmonary injury. Hence, EV-*let-7a-5p* hold promise for improving adverse lung conditions associated with ALI. The illustration was created with BioRender.com

EVs provide significant advantages, such as high biocompatibility, safety, cell penetration, and biodegradability, making them natural carriers for therapeutic RNAs. However, the development of effective cargo-loading methods is crucial for advancing their therapeutic potential. This study successfully enriched EVs with *let-7a-5p* miRNA using the CNP platform. These engineered *let-7a-5p* miRNA-encapsulated EVs at a 400-fold higher concentration demonstrated immunomodulatory and tissue repair capabilities, which hold promise for treating ALI. In conclusion, *let-7a-5p*-enriched EVs demonstrated a significant potential for treating ALI/ARDS (Fig. 6). However, notably, the lack of clinical trials limits the scope of the present study. Further research and clinical investigations are warranted to validate the safety and efficacy of this approach, emphasizing the need for future efforts in this direction.

### Supplementary Information

**Additional file 1: Figure S1.** Validation of MSC surface markers and their differentiation ability.** Figure S2.** Characterization of the exosomal surface markers of EVs via ExoView.** Figure S3.** RNA content determined by qRT-PCR.** Figure S4.** EV uptake by lung-originated cell lines.** Table S1.** The total concentration, mean, and mode size of EVs from three batches.** Table S2.** Predicted lung fibrosis-related targets of* let-7a-5p*.** Table S3.** Top 20* let-7a-5p* mediated pathways analyzed via CPDB.

## Data Availability

The datasets used and/or analyzed in the current study are available from the corresponding author upon reasonable request.

## Abbreviations

ALI: Acute lung injury
EVs: Extracellular vesicles
WJ-MSCs: Wharton’s jelly-mesenchymal stem cells
CNP: Cellular nanoporation
TEM: Transmission electron microscope
NTA: Nanoparticle tracking analysis
TIRF: Total internal reflection fluorescence
TFF: Tangential flow filtration
ISEV: International society for extracellular vesicles
ARDS: Acute respiratory distress syndrome
HUC-MSCs: Human umbilical cord mesenchymal stem cells
SaO_2_: Oxygen saturation
