# Supplementary material for: Engineered extracellular vesicles carrying let-7a-5p for alleviating inflammation in acute lung injury
Source: J Biomed Sci. 2024 Mar 19;31:30. doi: 10.1186/s12929-024-01019-4 (PMC10949767; doi:10.1186/s12929-024-01019-4)
Supplement: Supplementary file 1 — Additional file 1: Figure S1. Validation of MSC surface markers and their differentiation ability. Figure S2. Characterization of the exosomal surface markers of EVs via ExoView. Figure S3. RNA content determined by qRT-PCR. Figure S4. EV uptake by lung-originated cell lines. Table S1. The total concentration, mean, and mode size of EVs from three batches. Table S2. Predicted lung fibrosis-related targets of let-7a-5p. Table S3. Top 20 let-7a-5p mediated pathways analyzed via CPDB. [file 12929_2024_1019_MOESM1_ESM.pdf]

Additional files: Supplementary Information

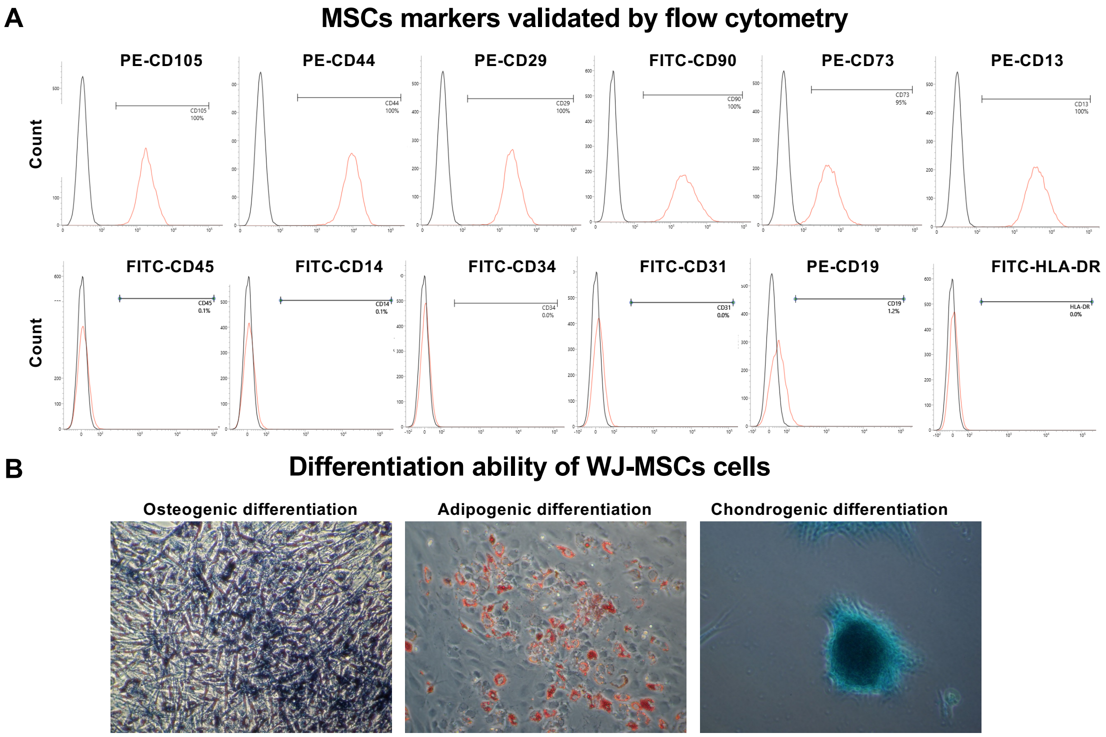

Additional file 1: Figure S1. Validation of MSC surface markers and their differentiation ability.

(**A**) Validation of MSC markers using flow cytometry, including both positive and negative markers for MSCs. (**B**) Validation of the differentiation ability of WJ-MSCs through staining for three different mesenchymal lineages.

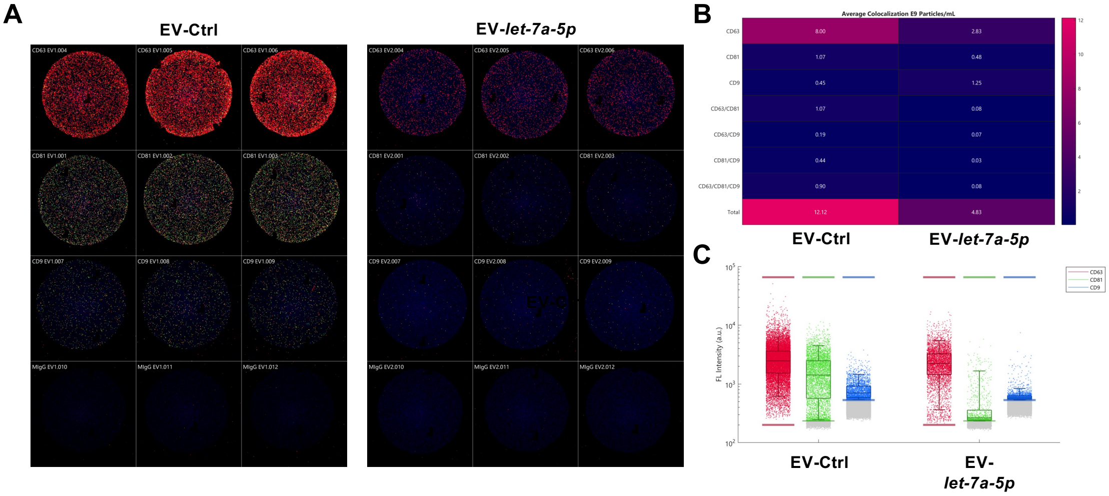

Additional file 2: Figure S2. Characterization of the exosomal surface markers of EVs via ExoView.

(**A**) The EV signal was detected by a single particle interferometric reflectance imaging sensor via ExoView. (**B**) Colocalization analysis was performed using ExoView. We used anti-CD63 as a capture probe for EVs. (**C**) Fluorescence box plots for CD63 captured EVs.

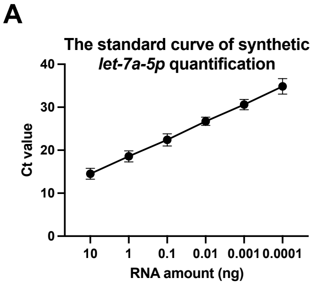

Additional file 3: Figure S3. RNA content determined by qRT-PCR.

(**A**)The standard curve quantification of synthetic *let-7a-5p* was measured by qPCR.

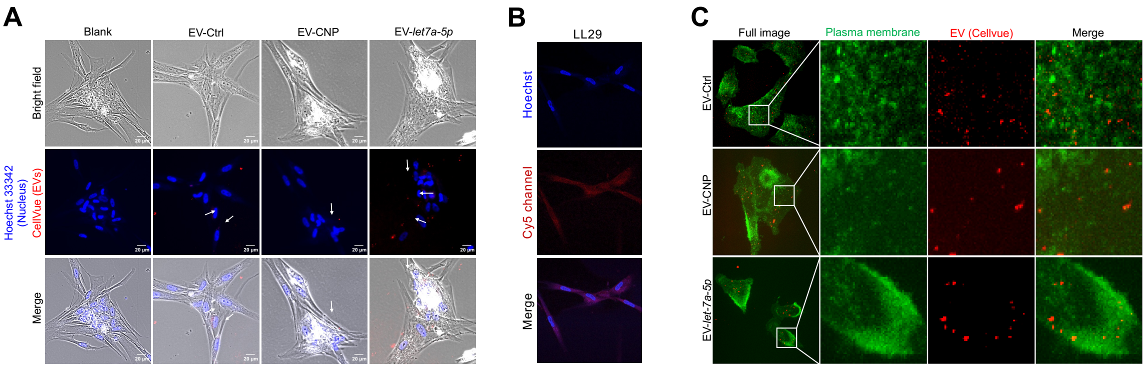

Additional file 4: Figure S4. EV uptake by lung-originated cell lines.

(**A**) EVs were uptake by LL29 cells. The image was taken with an epi-fluorescence microscope. (B) A confocal microscope was used to evaluate the single EV uptake. However, the remarkable autofluorescence background was demonstrated by LL29 cells. (C) Dynamics of single-EV analysis was performed in A549 cells. The images were taken using Total Internal Reflection Fluorescence microscopy (TIRFM, Nikon).

Additional file 5: Table S1. The total concentration, mean, and mode size of EVs from three batches

The total concentration, mean, and mode size of EVs were calculated for naive EV (EV-Ctrl), EV-CNP (cellular electroporation with PBS as vehicle control), and *let-7a-5p*-enriched EVs (cellular electroporation with *let-7a-5p* plasmid). The results are representative of n = 3 biologically independent experiments (3 batches). Data were presented as mean ± standard deviation.

WJ-MSCs					
		Batch 1	Batch 2	Batch 3	Average
**EV-Ctrl**	Concentration (particles/mL)	1.16E+08	1.38E+08	1.61E+08	1.38E+08 ± 2.25E+07
	Mean size (nm)	198.6	183.7	130.9	171.1 ± 35.6
	Mode size (nm)	123.5	140.5	93.7	119.2 ± 23.7
**EV-CNP**	Concentration (particles/mL)	8.50E+08	7.41E+08	8.29E+08	8.07E+08 ± 5.78E+07
	Mean size (nm)	105.0	100.6	101.0	102.2 ± 2.4
	Mode size (nm)	73.9	78.4	73.1	75.1 ± 2.9
**EV-*let-7a-5p***	Concentration (particles/mL)	7.01E+08	8.90E+08	1.03E+09	8.74E+08 ± 1.65E+08
	Mean size (nm)	116.8	124.0	144.4	128.4 ± 14.3
	Mode size (nm)	84.7	85.1	96.0	79.6 ± 9.2

Additional file 6: Table S2. Predicted lung fibrosis-related targets of *let-7a-5p*

The listed targets were ranked based on the target scores calculated by miRDB, and this table only shows the targets related to lung fibrosis.

Target	Target Score	Gene Description
COL3A1	95	collagen type III alpha 1 chain
COL1A2	92	collagen type I alpha 2 chain
COL4A2	92	collagen type IV alpha 2 chain
FNDC3A	89	fibronectin type III domain containing 3A
COL27A1	88	collagen type XXVII alpha 1 chain
COL4A1	88	collagen type IV alpha 1 chain
COL4A3BP	87	collagen type IV alpha 3 binding protein
COL4A6	87	collagen type IV alpha 6 chain
FNDC3B	81	fibronectin type III domain containing 3B
COL5A2	80	collagen type V alpha 2 chain
LRFN4	79	leucine rich repeat and fibronectin type III domain containing 4
IL10	76	interleukin 10
TAB2	72	TGF-beta activated kinase 1 (MAP3K7) binding protein 2
COL14A1	62	collagen type XIV alpha 1 chain
COL1A1	57	collagen type I alpha 1 chain
CTHRC1	54	collagen triple helix repeat containing 1
COL9A1	52	collagen type IX alpha 1 chain
SMAD2	50	SMAD family member 2

Additional file 7: Table S3. Top 20 *let-7a-5p* mediated pathways analyzed via CPDB

A total of 990 targets were obtained from miRDB, followed by a query in CPDB for pathway analysis, highlighting pathways related to collagen synthesis.

Rank	Pathway name	p-value	q-value	pathway source
1	Angiopoietin Like Protein 8 Regulatory Pathway	1.00E-06	0.0019	Wikipathways
2	AGE-RAGE signaling pathway in diabetic complications - Homo sapiens (human)	3.47E-06	0.0033	KEGG
3	Hepatitis C and Hepatocellular Carcinoma	2.42E-05	0.0139	Wikipathways
4	Scavenging by Class A Receptors	2.91E-05	0.0139	Reactome
5	FoxO signaling pathway - Homo sapiens (human)	4.63E-05	0.0177	KEGG
6	Collagen formation	6.52E-05	0.0185	Reactome
7	Collagen chain trimerization	6.76E-05	0.0185	Reactome
8	Insulin Signaling	8.44E-05	0.0193	Wikipathways
9	EGF-Core	9.97E-05	0.0193	Signalink
10	Type II diabetes mellitus - Homo sapiens (human)	0.000101	0.0193	KEGG
11	Insulin-like Growth Factor-2 mRNA Binding Proteins (IGF2BPs/IMPs/VICKZs) bind RN	0.00014	0.0244	Reactome
12	Collagen biosynthesis and modifying enzymes	0.000154	0.0245	Reactome
13	PI3K-Akt signaling pathway	0.000187	0.0248	Wikipathways
14	SHP2 signaling	0.000199	0.0248	PID
15	MAPK Signaling Pathway	0.0002	0.0248	Wikipathways
16	Bladder cancer - Homo sapiens (human)	0.000207	0.0248	KEGG
17	Prolactin signaling pathway - Homo sapiens (human)	0.000236	0.0266	KEGG
18	Transcriptional Regulation by VENTX	0.000294	0.0296	Reactome
19	O-linked glycosylation	0.000294	0.0296	Reactome
20	MAPK signaling pathway - Homo sapiens (human)	0.000327	0.0303	KEGG
